# Refeeding Syndrome and Its Related Factors in Critically Ill Coronavirus Disease 2019 Patients: A Prospective Cohort Study

**DOI:** 10.3389/fnut.2022.830457

**Published:** 2022-04-11

**Authors:** Zahra Vahdat Shariatpanahi, Maryam Vahdat Shariatpanahi, Erfan Shahbazi, Shaahin Shahbazi

**Affiliations:** ^1^Department of Clinical Nutrition and Dietetics, Faculty of Nutrition Sciences and Food Technology, National Nutrition and Food Technology Research Institute, Shahid Beheshti University of Medical Sciences, Tehran, Iran; ^2^Department of Psychiatry, Faculty of Medicine, Azad University of Medical Sciences, Tehran, Iran; ^3^School of Medicine, Tehran University of Medical Sciences, Tehran, Iran; ^4^Department of Gastroenterology, Faculty of Medicine, Ilam University of Medical Sciences, Ilam, Iran

**Keywords:** malnutrition, GLIM, muscle loss, protein intake, calorie intake

## Abstract

**Background and Aim:**

Malnutrition and its complications is usually neglected in critically ill COVID-19 patients. We conducted the present study to investigate the prevalence of refeeding syndrome and its related factors in this group of patients.

**Methods:**

In this prospective cohort study, 327 patients were assessed for being at risk and developing refeeding syndrome. The criteria was ASPEN consensus recommendations for refeeding syndrome released in 2020. Malnutrition was assessed based on global leadership initiative on malnutrition (GLIM) criteria. The relation between actual protein, calorie intake, and refeeding syndrome was also evaluated *via* cox regression model. The data concerning calorie and protein intake were gathered for 5 days after initiating feeding. The daily protein and calorie intake were divided by kilogram body weight in order to calculate the actual protein (g/kg/day) and energy (kcal/kg/day) intake.

**Results:**

Among the subjects, 268 (82%) were at risk of refeeding syndrome and 116 (36%) got involved in this syndrome. Malnutrition, according to the GLIM criteria, was found in 193 (59%) of the subjects. In the at-risk population, the risk of refeeding syndrome was reduced by 90% with the rise in protein intake (CI; 0.021–0.436, *P* = 0.002), increased by 1.04 times with the increase in age (CI; 1.032–1.067, *P* < 0.001), and by 1.19 times with the rise in the days from illness onset to admission (CI; 1.081–1.312, *P* < 0.001) in adjusted cox model analysis.

**Conclusion:**

The incidence of refeeding syndrome is relatively high, which threatens the majority of critically ill COVID-19 patients. Increased protein intake was found to reduce the occurrence of refeeding syndrome.

## Introduction

Coronavirus disease 2019 (COVID-19) infection, primarily identified in December 2019, strongly affects the patient’s nutritional status. COVID-19-infected patients are considered to be at a high risk of malnutrition (1). This risk particularly increases in severe and critical cases. Several factors could be behind a malnourished state; inflammation and hypercatabolism, anorexia, depression, anxiety, Hypoguesia, Hyposmia, immobilization, digestive discomforts (nausea, diarrhea, and pain), and comorbidity all lead to a malnourished state with loss of lean body mass and negative nitrogen balance (2, 3).

One of the complications associated with the initiation of feeding in malnourished patients is refeeding syndrome. Refeeding syndrome, a life-threatening condition, is a metabolic disorder characterized by electrolyte disturbances and shifting fluid following reintroducing oral, enteral, and parenteral nutrition in malnourished patients (4). It is well known that malnutrition is prevalent in COVID-19 infected patients, especially in severe and critically ill ones (5). Unfortunately, a big proportion of hospital staff are not informed about the importance of malnutrition and the precautions needed to initiate feeding. In COVID-19 cases, in particular, nutrition therapy is neglected on a number of occasions due to the special condition of this disease. Therefore, it is necessary to be careful about the risk of developing refeeding syndrome. Since there is no universally accepted definition for refeeding syndrome, the data in this regard are controversial (4). In a study conducted on critically ill patients, using the hypophosphatemia as the diagnostic criteria for refeeding syndrome, its incidence was reported to be 34% (6). Additionally, in a prospective cohort study on patients hospitalized both in ward and intensive care unit (ICU), this rate was 2% based on the following diagnostic criteria: severely low electrolytes (potassium, magnesium, and phosphorus), fluid overload, and disturbance of organ function (7). Recently, the American Society for Parenteral and Enteral Nutrition (ASPEN) consensus recommendations for refeeding syndrome have been released (4). Herein, we used these recommendations to evaluate the incidence of refeeding syndrome in critically ill COVID-19 patients and investigate its association with calorie and protein intake.

## Materials and Methods

### Study Design and Participants

**FIGURE 1:**
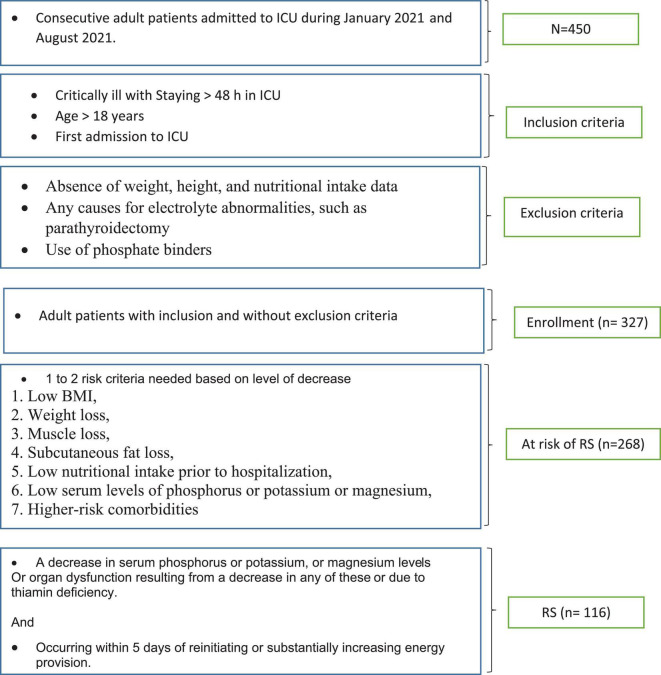
Flowchart of patients.

The current prospective cohort study was conducted from January 2021 to August 2021. The study was approved by the Ilam University of Medical Sciences Ethics Committee (IR.MEDILAM.REC.1399.270) and informed consent was obtained from patients. Critically ill adult patients with the age of 18 years and older, who were sequentially hospitalized in ICU with positive real-time fluorescence polymerase chain reaction (RT-PCR) for COVID-19, were included in the study. The critically ill subjects were defined as those with respiratory failure, shock, or multiorgan dysfunction, who should have been treated in ICU (8). The exclusion criteria were the absence of data concerning weight, height, and nutritional intake, in addition to any causes for electrolyte abnormalities, such as parathyroidectomy or use of phosphate binders. In the patients with a readmission during the study period, the data from the first admission were considered for analysis (Figure 1).

### Data Collection

During the first 48 h of admission, prior to edema formation, anthropometric measurements were done to assess malnutrition and the risk of refeeding syndrome. The ASPEN consensus recommendation criteria were used to determine whether the patients were at risk of refeeding syndrome (4). The collected data were: (1) low BMI, (2) percentage of weight loss, (3) muscle loss, (4) subcutaneous fat loss, (5) low nutritional intake prior to hospitalization, (6) low serum levels of electrolytes (phosphorus, potassium, and magnesium), and (7) higher-risk comorbidities. ASPEN criteria added the physical exam findings, including loss of subcutaneous fat and muscle mass, to the previous National Institute for Health and Care Excellence (NICE) criteria.

Feeding was started during the first 48 h of admission to the ICU. After feeding, the patients were followed for the occurrence of refeeding syndrome. Refeeding syndrome is defined by ASPEN Consensus Recommendations as: (1) a decrease in any 1, 2, or 3 of serum phosphorus, potassium, and/or magnesium levels and/or organ dysfunction as a result of a decrease in any of these and/or due to thiamine deficiency, and (2) occurring within 5 days of reinitiating or substantially increasing energy provision.

Hypokalemia, hypophosphatemia, and hypomagnesemia were defined as the serum levels less than 3.5 mg/dL (3.5 mmol/L), 2.5 mg/dL (0.8 mmol/L), and 1.46 mg/dL (0.6 mmol/L), respectively, based on hospital reference for adults. Moreover, the patients were observed on a daily basis for any symptoms of hypokalemia, hypomagnesemia, hypophosphatemia, and thiamine deficiency.

Malnutrition was assessed by Global Leadership Initiative on Malnutrition (GLIM) criteria. The data about calorie and protein intake were obtained from the medical records during hospitalization. Furthermore, calories from propofol or any macronutrient infusion were recorded for each patient. As refeeding syndrome occurs within 5 days of reinitiating or substantially increasing energy provision, the mean calorie and protein intake were calculated for 5 days after starting feeding in each patient for statistical analysis. If refeeding syndrome occurred in any patients earlier than 5 days, the average calorie intake was limited to the days before getting the syndrome. To calculate the actual protein intake (g/kg/day), daily protein intake was divided by kilogram body weight. In addition, the daily calorie intake was divided by kilogram body weight to obtain the actual energy (kcal/kg/day) intake.

To calculate non-protein calorie to nitrogen ratio (NPC: N), the mean calorie of protein intake was subtracted from the mean total energy intake, and then divided by the mean nitrogen intake. Nitrogen intake was estimated by dividing the actual protein intake to 6.25.

### Outcome Measures

The primary outcome measure was the incidence of refeeding syndrome. The secondary outcome measure was the relation between calorie and protein intake with the occurrence of refeeding syndrome.

### Statistical Analysis

The data were analyzed through the use of SPSS software version 22.0 (Statistical Package for the Social Sciences, IBM Corp., Armonk, NY, United States). *P*-values equal to or less than 0.05 were considered as the level of significance. Kolmogorov-smirnov (K-S) test was conducted to examine the distribution normality of the variables. All the variables were normally distributed, so the continuous variables were regarded as means ± standard deviation (SD). The categorical data were presented as proportions (number and percentage).

In the patients at risk of refeeding syndrome, the Cox regression analysis was utilized to estimate the hazard ratio (HR) of refeeding syndrome with the actual calorie and protein intake and NPC: N with and without adjustments for confounder variables with *P* < 0.05, including, age, comorbidity, and the number of days from illness onset to admission.

## Results

### Characteristics of the Population

A total of 327 patients were included in the study. Among these patients, 268 (82%) were at risk of refeeding syndrome. Overall, 116 subjects (36%) developed refeeding syndrome and 158 (47%) died. Moreover, 193 (59%) of the critically ill COVID-19 patients were malnourished based on GLIM criteria.

### Characteristics of the Patients at Risk of Refeeding Syndrome

Table 1 shows the demographic and clinical characteristics of the patients who were at risk of refeeding syndrome. Among the criteria in the ASPEN concerning the risk of refeeding syndrome, the highest prevalence of the syndrome belonged to reduced food intake prior to hospitalization, followed by weight loss, low BMI, fat/muscle loss, and electrolyte disturbances.

**TABLE 1:** Demographic and clinical characteristics of at risk patients, stratified by Refeeding syndrome.

Variable	At risk for RS, total (*n* = 268)	Non- RS (*n* = 152)	RS (*n* = 116)	*P*-value
Age, Year	61.04 ± 12.36	56.80 ± 11.51	66.59 ± 11.22	<0.001
Male	139 (52)	84 (55)	55 (47)	0.20
APACHE II	16.05 ± 2.90	15.98 ± 2.69	16.11 ± 3.06	0.71
Weight (Kg)	71,76 ± 12.90	72.35 ± 13.73	71.00 ± 11.72	0.39
Comorbidity	113 (42)	53 (35)	60 (52)	0.006
Malnutrition (GLIM)	193 (72)	96 (63)	97 (84)	<0.001
Reduced food intake prior hospitalization	225 (84)	109 (72)	116 (100)	<0.001
Anorexia/weakness	213 (80)	97 (64)	116 (100)	<0.001
Weight loss	169 (63)	72 (47)	97 (84)	<0.001
Low BMI	35 (13)	8 (5)	27 (23)	<0.001
Muscle mass loss	20 (8)	8 (5)	12 (10)	0.11
Subcutaneous fat loss	68 (25)	28 (18)	40 (34)	0.002
Baseline potassium	4.10 ± 0.59	4.13 ± 0.62	4.08 ± 0.54	0.47
Baseline phosphate	3.05 ± 0.62	3.09 ± 0.65	3.01 ± 0.60	0.29
Baseline magnesium	1.80 ± 0.34	1.83 ± 0.37	1.77 ± 0.31	0.15
Medication				
Antiviral	268 (100)	152 (100)	116 (100)	1
Antibiotic	154 (57)	74 (49)	80 (69)	0.001
Glucocorticoid	268 (100)	152 (100)	116 (100)	1
Days from illness onset to admission	7.89 ± 1.72	7.57 ± 1.54	8.24 ± 1.86	0.002
Admission from				0.005
Emergency	140 (52)	68 (45)	72 (62)	
Ward	128 (48)	84 (55)	44 (38)	
Mortality	126 (47)	41 (27)	85 (73)	<0.001

*RS, Refeeding syndrome; APACHE, Acute Physiology and Chronic Health Evaluation; GLIM, Global Leadership Initiative on Malnutrition. Data are reported as X^2^ test (n,%) or T-test (mean, SD).*

193 (73%) at-risk patients were malnourished. 116 (43%) at-risk patients were involved in refeeding syndrome.

No differences were observed between the two groups concerning sex and APACHE II score. The mean age of the subjects in the group with refeeding syndrome was significantly higher than that in the group without refeeding syndrome. Furthermore, the rate of comorbidity and the average days from illness onset to admission were higher in those with refeeding syndrome.

Among the cases at risk of the syndrome, 126 (47%) died in the hospital; this rate was twice as high as that in the patients with refeeding syndrome.

Table 2 shows the feeding characteristics of patients. The mean actual protein intake (g/kg/day) was significantly lower in those involved in refeeding syndrome in comparison with the patients without refeeding syndrome. There were no differences in the mean actual energy intake (Kcal/kg/day) and NPC: N between the two groups.

**TABLE 2:** Feeding characteristics of at risk patients, stratified by Refeeding syndrome.

Variable	At risk for RS, total (*n* = 268)	Non- RS (*n* = 152)	RS (*n* = 116)	*P*-value
Feeding start time (day)	1.48 ± 0.5	1.41 ± 0.49	1.57 ± 0.49	0.08
Type of feeding				
Enteral, oral	180 (67)	105 (69)	75 (65)	0.44
Parenteral (propofol, macronutrients)	88 (33)	47 (31)	41 (35)	
Mean actual protein intake (g/kg/day)	0.56 ± 0.11	0.59 ± 011	0.52 ± 0.11	<0.001
Mean actual energy intake, (Kcal/kg/day)	13.64 ± 2.87	13.42 ± 3.04	13.93 ± 2.62	0.15
NPC:N	125.62 ± 43.39	122.26 ± 44.92	130.02 ± 41.07	0.14

*RS, Refeeding syndrome; NPC: N; Non-protein Calorie to Nitrogen Ratio. Data are reported as X^2^ test (n,%) or T-test (mean, SD).*

To determine the relationship between food intake and refeeding syndrome, cox regression analysis was performed. The results revealed that with the increase in the actual protein intake (g/kg/day), hazard ratio of refeeding syndrome decreased by about 96% (Table 3). Similar to age, the days from illness onset to admission and comorbidity were significantly associated with refeeding syndrome in crude model (HR = 1.160, 1.062, 2.298, respectively). As shown in Table 3, adjusted cox regression analysis was employed for controlling these covariates. The results showed that hazard ratio of refeeding syndrome decreased by 90% with the increase in protein intake. Furthermore, hazard ratio of refeeding syndrome rose by about 1.05 times with the increase in age and by 1.19 times with the increase in the days from onset of illness before admission.

**TABLE 3:** Cox regression model for refeeding syndrome.

	HR	CI	*P*-value	Adjusted HR	CI	*P*-value
Mean actual protein intake (g/kg/day)	0.042	0.011–0.155	<0.001	0.095	0.021–0.436	0.002
Days before admission	1.160	1.050–1.281	0.003	1.191	1.081–1.312	<0.001
Age	1.062	1.046–1.080	<0.001	1.049	1.032–1.067	<0.001
Comorbidity	2.298	1.591–3.320	<0.001	1.156	0.788–1.695	0.45

There was no association between the mean actual energy intakes (Kcal/kg/day), NPC: N, and the occurrence of refeeding syndrome in cox regression analysis.

## Discussion

In the present study, which was conducted on 327 critically ill COVID-19 patients, 82% were at risk of refeeding syndrome and 36% were involved in refeeding syndrome. Refeeding syndrome was developed only in the patients who were at risk of refeeding syndrome. In other words, 43% of the at-risk subjects were involved in refeeding syndrome. On the other hand, the highest rate of mortality was observed in the patients with refeeding syndrome, in a way that among 158 dead cases, 85 had refeeding syndrome, 41 were at risk of refeeding syndrome, and 32 were in none of these two groups. Another interesting finding was that with the increase in protein intake, the risk of refeeding syndrome was reduced by 90% in the at-risk population. This association was not seen between the syndrome and energy and NPC: N. Increased age and days from illness onset to admission had also a minor association with refeeding syndrome.

To the best of our knowledge, there has been no reports of the prevalence of refeeding syndrome in critically ill COVID-19 patients to date. However, ASPEN recommends that in critically ill COVID-19 patients, trophic nutrition be started and slowly increased to a full dose of 15 to 20 kcal/kg in the first week to prevent refeeding syndrome (9). Furthermore, there is no universal definition for refeeding syndrome. The current study applied recent ASPEN consensus recommendation criteria to detect the critically ill COVID-19 patients who were at risk of refeeding syndrome or suffering from it. The reported prevalence of refeeding syndrome in other diseases is variable and underestimated at ICU admission as reported in literature review (10). In a study conducted on 178 patients, who acutely admitted to the department of internal medicine, applying the NICE criteria and taking hypophosphataemia as the main indicator for the presence of this syndrome, 14% developed refeeding syndrome and 54% were considered to be at risk of the syndrome (11). In a retrospective study in a neurocritical care unit, 328 neurocritically ill patients who received total enteral nutrition were assessed for refeeding syndrome. The occurrence of refeeding syndrome was 17.1% based on low phosphate level (12). Valizade et al. reported that the incidence of refeeding syndrome was 21.4% in a general critical care unit with no definition for refeeding syndrome diagnosis (13). In our study, the reason behind the higher incidence of both at-risk and involved cases in refeeding syndrome was the use of a more comprehensive tool for the assessment.

In the present work, 72% of the at-risk patients had malnutrition based on GLIM criteria. In fact, the criteria defined by ASPEN consensus recommendation for the risk of refeeding syndrome are similar to those for assessment of malnutrition by GLIM criteria (14). The only difference is the item of prefeeding serum electrolyte disturbances, which is specific to the at risk for refeeding syndrome criteria. This finding showed the importance of conducting a nutritional assessment in critically ill COVID-19 patients *via* a comprehensive tool, such as GLIM, to find those at risk of refeeding syndrome.

This manuscript also demonstrated the tight relationship between the amount of protein intake and the prevention of refeeding syndrome. ASPEN consensus recommendations for avoidance and treatment of refeeding syndrome in at-risk adults have not recommended an appropriate amount of protein intake at this time (4). Additionally, regarding calorie intake, it has recommended initiating with 10–20 kcal/kg for the first 24 h with gradually increasing it. In our research, the mean calorie intake was 13.58 ± 2.83 in the at-risk patients and there was no significant difference between the two groups. This amount of calorie is in agreement with ASPEN recommendations, which is the reason behind non-association between calorie intake and refeeding syndrome in our study. We also found that older patients and those who spent more days from illness onset to admission were more involved in refeeding syndrome.

The mean NPN: N ratio was 125.6 in the total population at risk of refeeding syndrome. This value was higher in the patients with refeeding syndrome although the difference was not significant. It was demonstrated that in the critically ill patients, an NPC: N ratio of 100:1 or less may be optimal (15). Most enteral formulas have a high NPC: N ratio; therefore, considering protein supplementation beside the formula intake may be of value in these patients.

Our investigation had certain strengths and limitations. The incidence of refeeding syndrome in critically ill COVID-19 patients and its association with calorie and protein intake were studied herein for the first time. Unfortunately, it was a single-center study and due to the lack of previous research on this topic, the sample size was not calculated. Moreover, the data concerning the height and weight of certain patients were self-reported by the patients or caregivers.

## Conclusion

The current study indicated that most critically ill COVID-19 patients are at risk of refeeding syndrome. The incidence of refeeding syndrome remained relatively high despite the decreased calorie intake. Increased protein intake was found to reduce the incidence of this syndrome.

As most enteral formulas have a high NPC: N ratio, addition of modular protein supplements for critically ill COVID-19 patients should be considered. Evaluation of malnutrition at admission is valuable to detect patients at risk of refeeding syndrome. Furthermore, with the increase in age and the days from onset of illness to admission, the risk of refeeding syndrome augments.

## Data Availability Statement

The datasets used and analyzed during this study are available from the corresponding author on reasonable request.

## Ethics Statement

The studies involving human participants were reviewed and approved by IR.MEDILAM.REC.1399.270. The patients/participants provided their written informed consent to participate in this study.

## Author Contributions

SS, ZV, MV, and ES performed the material preparation and data collection and analysis. ZV wrote first draft of the manuscript. All authors commented on previous versions of the manuscript, read and approved the final manuscript, and contributed to the study conception and design.

## Conflict of Interest

The authors declare that the research was conducted in the absence of any commercial or financial relationships that could be construed as a potential conflict of interest.

## Publisher’s Note

All claims expressed in this article are solely those of the authors and do not necessarily represent those of their affiliated organizations, or those of the publisher, the editors and the reviewers. Any product that may be evaluated in this article, or claim that may be made by its manufacturer, is not guaranteed or endorsed by the publisher.

## Abbreviations

ASPEN: American Society of parenteral and enteral nutrition
APACHE: Acute Physiology and Chronic Health Evaluation
BMI: Body Mass Index
COVID-19: Coronavirus disease 2019
GLIM: global leadership initiative on malnutrition
ICU: Intensive care unit; to Nitrogen Ratio
RT-PCR: Real-time fluorescence polymerase chain reaction
RS: Refeeding syndrome.
